# Supplementary material for: Detecting Collagen by Machine Learning Improved Photoacoustic Spectral Analysis for Breast Cancer Diagnostics: Feasibility Studies With Murine Models
Source: J Biophotonics. 2024 Nov 26;18(1):e202400371. doi: 10.1002/jbio.202400371 (PMC11700697; doi:10.1002/jbio.202400371)
Supplement: Supplementary file 1 — Figure S1. Characterizing collagen content of the cancerous tissues of three molecular breast cancer subtypes: triple negative (n = 18), HER2 (n = 19), and luminal (n = 13). The statistical analysis was assessed using the Kruskal–Wallis test and Dunn’s multiple comparison tests. Figure S2. Laser fluence for photoacoustic detection over the wavelengths of 1200–1700 nm. Figure S3. Principal component analysis scores of all samples (n = 100) on the two extracted latent variables (LVs) with eigenvalues greater than one. Figure S4. Characterizing collagen and lipid content in cancerous tissues through Masson’s trichome and H&E staining. (a) Statistical analysis (n = 50, ****p < 0.0001). (b) Representative staining images. The statistical analysis was assessed using the t‐test. [file JBIO-18-e202400371-s001.docx]

Supporting Information

Detecting collagen by machine learning improved photoacoustic spectral analysis for breast cancer diagnostics: feasibility studies with murine models

Jiayan Li, Lu Bai, Yingna Chen, Junmei Cao, Jingtao Zhu, Wenxiang Zhi^*^, Qian Cheng*


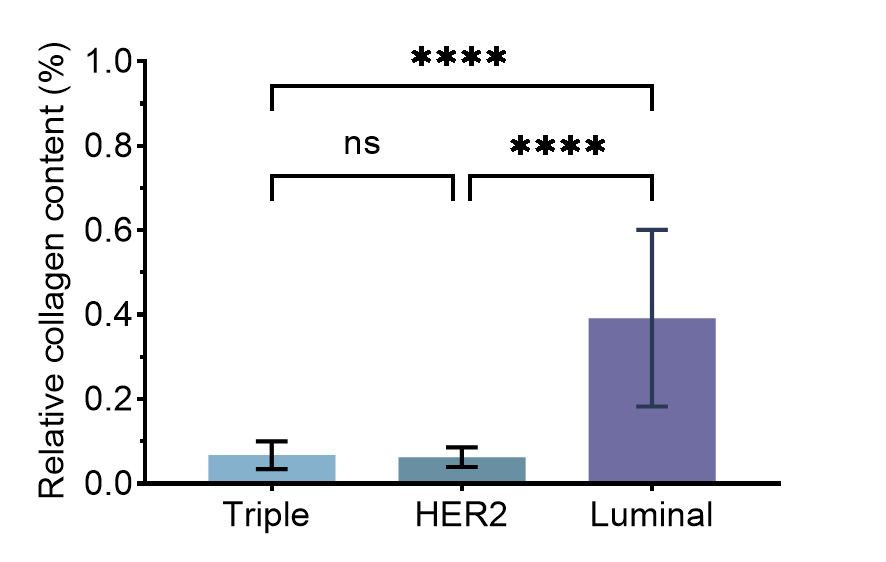


**Figure S1**. Characterizing collagen content of the cancerous tissues of three molecular breast cancer subtypes: triple negative (n=18), HER2 (n=19), and luminal (n=13). The statistical analysis was assessed using the Kruskal-Wallis test and Dunn’s multiple comparison tests.


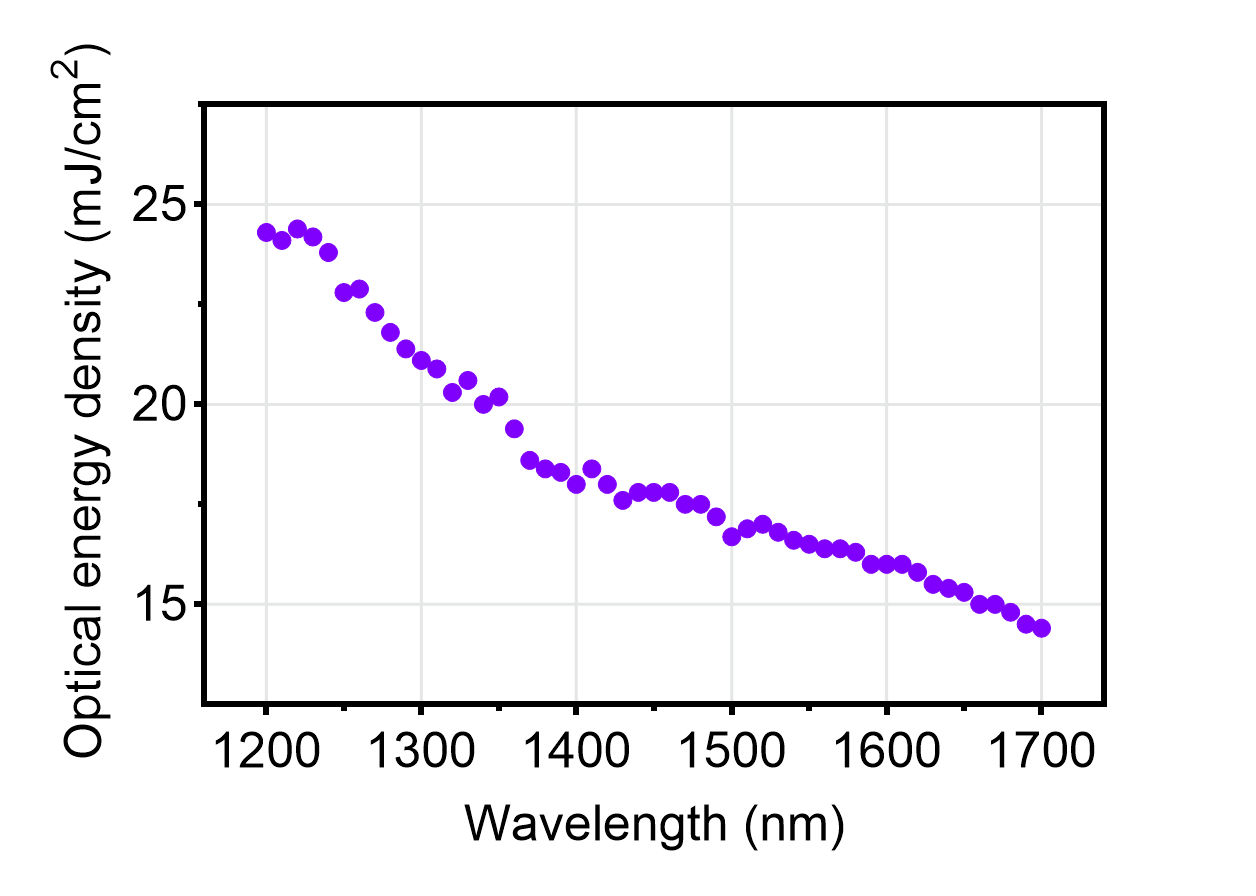


**Figure S2.** Laser fluence for photoacoustic detection over the wavelengths of 1200–1700 nm.


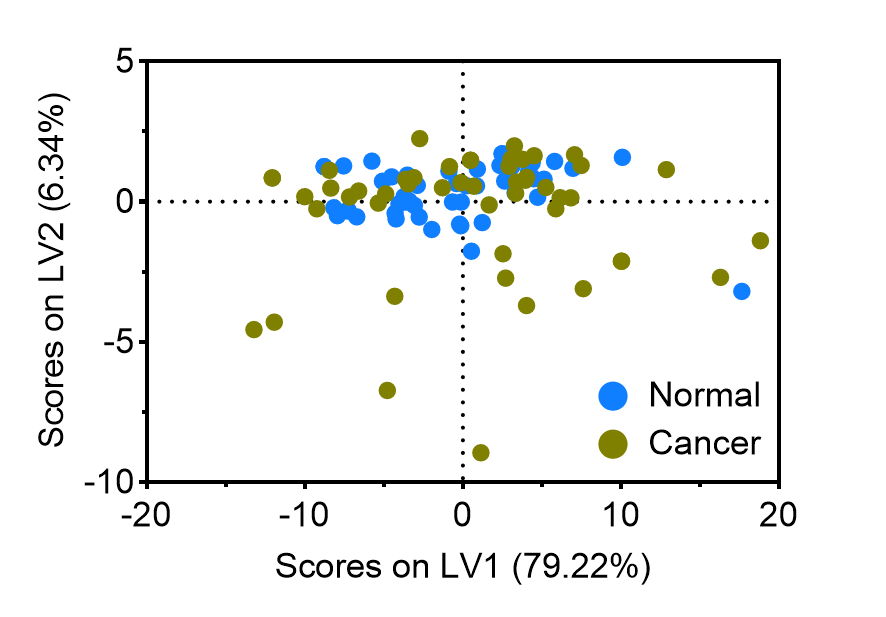


**Figure S3.** Principal component analysis scores of all samples (n=100) on the two extracted latent variables (LVs) with eigenvalues greater than one.


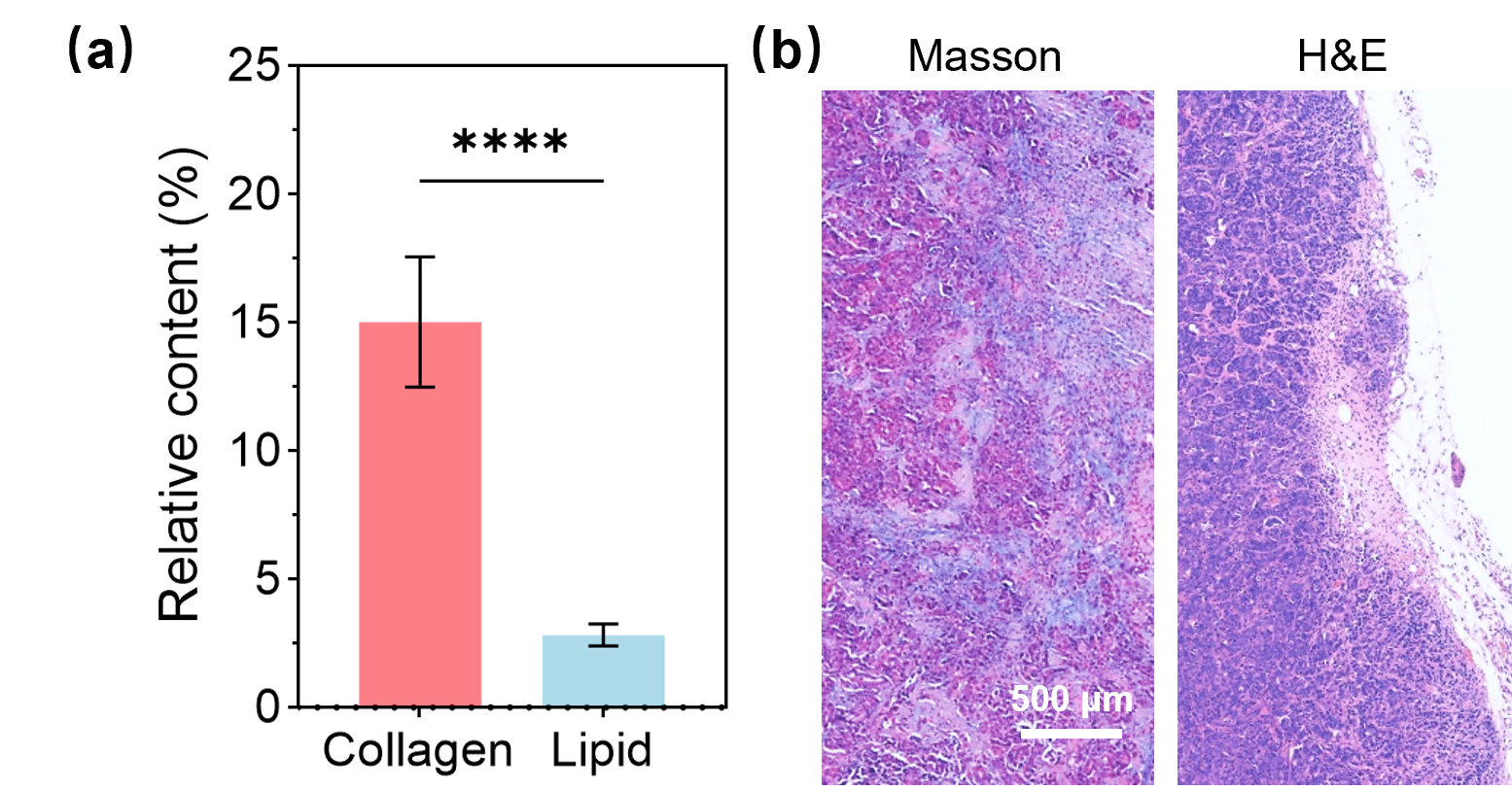


**Figure S4.** Characterizing collagen and lipid content in cancerous tissues through Masson’s trichome and H&E staining. (a) Statistical analysis (n=50, ****p<0.0001). (b) Representative staining images. The statistical analysis was assessed using the t-test.
